# Long noncoding RNA HULC promotes hepatocellular carcinoma progression

**DOI:** 10.18632/aging.102378

**Published:** 2019-10-23

**Authors:** Hongwei Zhang, Zhibin Liao, Furong Liu, Chen Su, He Zhu, Yani Li, Ran Tao, Huifang Liang, Bixiang Zhang, Xuewu Zhang

**Affiliations:** 1 Hepatic Surgery Center, Tongji Hospital, Tongji Medical College, Huazhong University of Science and Technology, Wuhan 430030, Hubei, China; 2 Clinical Medical Research Center of Hepatic Surgery at Hubei Province, Wuhan 430030, Hubei, China

**Keywords:** HULC, miR-2052, MET, hepatocellular carcinoma, long noncoding RNA

## Abstract

Long noncoding RNAs (lncRNAs) are overexpressed in many types of cancers, suggesting they may promote tumorigenesis. The lncRNA “highly upregulated in liver cancer” (HULC) promotes hepatocellular carcinoma (HCC) by mechanisms that are not fully understood. In the present study, we showed that HULC is overexpressed in HCC tissues, which correlates with an unfavorable prognosis in HCC patients. We also found that HULC promotes the proliferation, migration, and invasion of HCC cells *in vitro*, and xenograft tumor growth *in vivo*. Our mechanistic studies showed that HULC works as a competing endogenous RNA for miR-2052, and that the MET receptor tyrosine kinase is a downstream target of miR-2052 in HCC. Furthermore, HULC inhibits miR-2052, thereby stimulating MET expression in HCC. Finally, MET overexpression reverses the effects of HULC depletion. In sum, our findings reveal a novel regulatory signaling cascade, the HULC/miR-2052/MET axis, which could potentially be exploited for therapeutic benefits in the treatment of HCC.

## INTRODUCTION

Hepatocellular carcinoma (HCC) is one of the most common malignancies in the world [1, 2]. Its complex pathogenesis is complex can have multiple causes including chronic hepatitis B or C infection, exposure to aflatoxin B1, alcoholic and nonalcoholic fatty diseases, among others [3, 4]. In spite of recent research uncovering many genes and pathways that promote liver cancer development, treatments and patient prognosis remain unsatisfactory.

Noncoding RNAs (ncRNAs) are ubiquitous in organisms and participate in various cellular processes whose dysregulation contributes to many diseases [5]. Long noncoding RNAs (lncRNAs) are noncoding RNAs (ncRNAs) with 200 or more nucleotides [6]. Dysregulation of lncRNAs have been shown to contribute to the development of multiple types of cancer by mechanisms that are not fully understood [7–14].

Long non-coding RNA HULC is upregulated in liver cancer by the action of cAMP responsive element binding protein (CREB) [15], thereby promoting the occurrence and development of HCC. Furthermore, HULC inhibits miR-9 mediated apoptosis in hepatocellular carcinoma cells [16]. Hepatitis B virus X (HBX) protein elevates HULC levels and suppresses p18, which contributes to HCC proliferation [17]. Indeed, HULC can act as a cancer biomarker [18, 19]. Collectively, previous studies have shown that HULC is involved in the development and progression of HCC cells; however, the underlying mechanisms remain unclear.

In this study, we show that lncRNA HULC is upregulated in human liver cancer. Moreover, HULC accelerated the malignant progression of HCC cells through the miR-2052/MET axis, revealing a novel mechanism underlying HULC’s contribution to HCC development.

## RESULTS

### HULC is overexpressed in HCC

**Figure 1:**
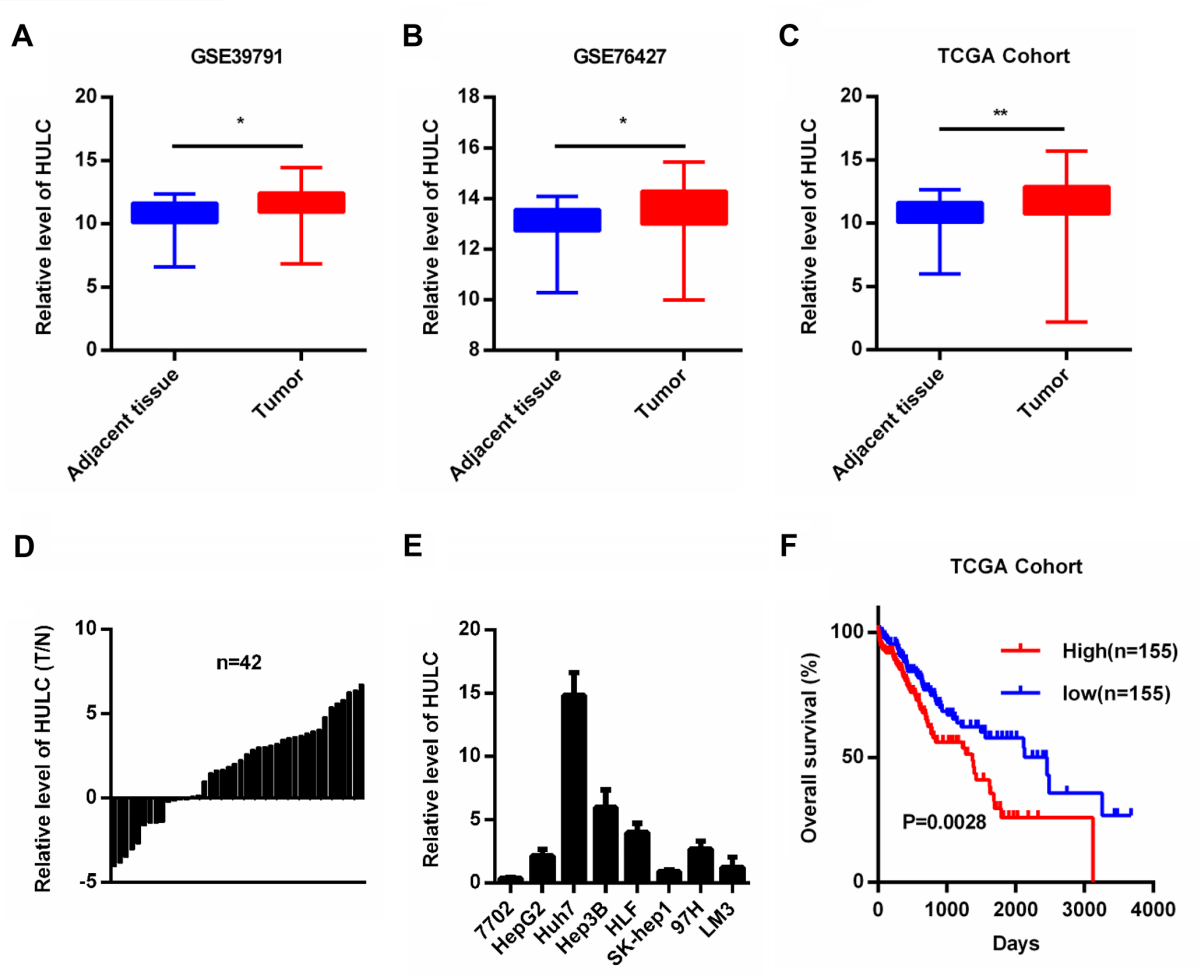
**HULC is overexpressed in HCC.** (**A**–**C**) HULC is upregulated in HCC tissues compared with adjacent non-tumor liver control tissues in two GEO data sets (GSE39791 and GSE76427) and a TCGA cohort. HULC expression in (**D**) HCC tissues (n=42) and (**E**) HCC cells analyzed by qPCR. (**F**) The overall survival of HCC patients from the TCGA cohort with high and low HULC expression. *P < 0.05, **P < 0.01.

To understand the role of HULC in HCC, we first analyzed HULC expression and HCC patient survival data from the public repositories. We observed higher levels of HULC in HCC tissues compared with controls (adjacent non-tumor liver tissue) in two Gene Expression Omnibus (GEO) datasets (GSE39791 and GSE76427), and The Cancer Genome Atlas (TCGA) cohort (Figure 1A–1C). In addition, we also measured HULC levels in 42 pairs of HCC tissues as well as their matched non-cancerous tissues. Our results showed increased expression of HULC in HCC tissues (Figure 1D). Next, we measured HULC levels in HCC cell lines (HepG2, Huh7, Hep3B, HLF, Sk-hep1, 97H, and LM3) and normal human hepatocyte (7702). HULC was overexpressed in HCC cell lines (Figure 1E). Taken together, these findings indicate that HULC is upregulated in HCC. To determine the prognostic potential of HULC in HCC patients, we generated Kaplan–Meier survival curves for the TCGA cohort. Our results showed that high levels of HULC correlated with poor overall survival (OS) of HCC patients (Figure 1F). These data suggested that HULC might promote tumorigenesis in HCC.

### HULC promotes proliferation, migration and invasion of HCC cells *in vitro*

**Figure 2:**
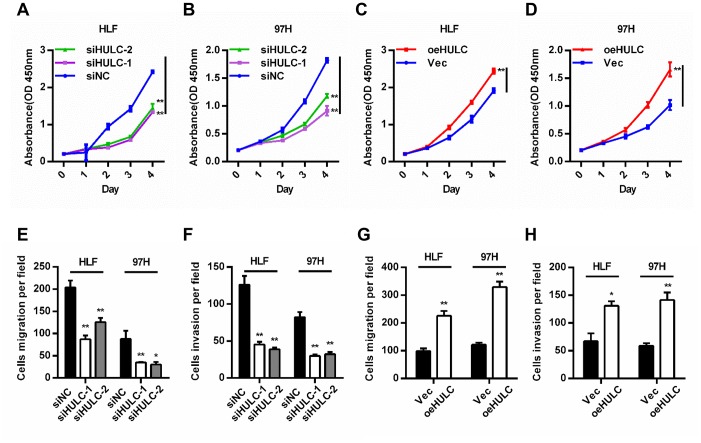
**HULC promotes proliferation, migration, and invasion of HCC cells *in vitro*.** (**A**–**D**) Viability of HCC cells transfected with siHULC or pcDNA-HULC as measured by CCK8 assays. (**E**–**H**) Transwell migration and invasion assays in cells with HULC silencing or overexpression. *P < 0.05, **P < 0.01.

HULC expression was silenced or overexpressed in HLF and 97H cells to investigate the biological function of HULC in HCC cells (Supplementary Figure 1A, 1B). CCK-8 assays indicated that downregulation of HULC led to decreased cell viability (Figure 2A, 2B), whereas overexpression of HULC efficiently enhanced cell viability (Figure 2C, 2D). Additionally, trans-well assays showed that HULC knockdown significantly reduced migration and invasion (Figure 2E, 2F and Supplementary Figure 1C), and overexpression of HULC contributed to migration and invasion of HLF and 97H cells (Figure 2G, 2H and Supplementary Figure 1D). The results above demonstrated that HULC promotes the proliferation, migration, and invasion of HCC cells.

### HULC is a ceRNA and acts as a sponge for miR-2052 in HCC cells

**Figure 3:**
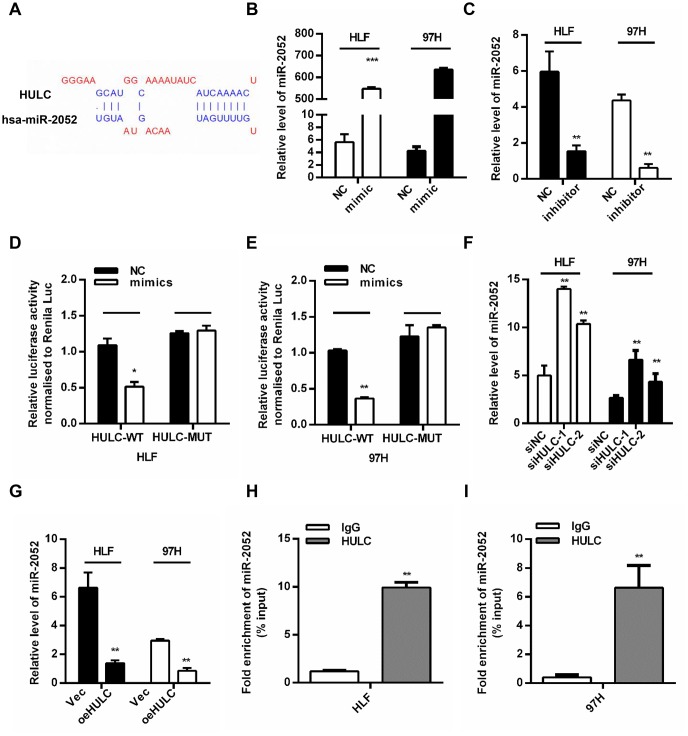
**HULC is a ceRNA and acts as a sponge for miR-2052 in HCC cells.** (**A**) The predicted binding sites of HULC and miR-2052. (**B**, **C**) Quantitative PCR analysis of miR-2052 after mimic and inhibitor transfection. (**D**, **E**) Relative luciferase activities of HULC-WT and HULC-MUT reporter measured in presence of miR-2052 mimic cotransfection. (**F**, **G**) qPCR analysis of miR-2052 expression in HCC cells transfected with siHULC or siNC, and Vec or pcDNA-HULC. (**H**, **I**) RIP assay was used to explore the enrichment of miR-2052 by HULC. *P < 0.05, **P < 0.01, ***P < 0.001.

By using bioinformatics predictions (DIANA Tools- LncBase Predicted v.2) [20], we found that HULC might be a ceRNA for miR-2052 (Figure 3A). We overexpressed miR-2052 through miR-2052 mimic transfection (Figure 3B) and silenced miR-2052 through miR-2052 inhibitor transfection (Figure 3C) in HLF and 97H cells. Luciferase reporter plasmid carrying the WT or MUT HULC sequence was cotransfected to measure luciferase activities. Our results showed that miR-2052 mimic transfection decreased the luciferase activity of HULC-WT reporter in HLF and 97H cells; however, mutation of the binding site abrogated this effect (Figure 3D, 3E). Moreover, HULC silencing increased miR-2052 levels (Figure 3F) while HULC overexpression decreased them (Figure 3G), indicating that HULC and miR-2052 inhibit each other’s expression. Additionally, to test whether HULC and miR-2052 engage in direct physical interactions, we performed RNA pull down followed by qPCR analysis. Our results showed that HULC interacted with miR-2052 directly (Figure 3H, 3I), indicating that HULC acts as a sponge for miR-2052 in HCC cells.

### miR-2052 inhibited the proliferation, migration and invasion of HCC cells *in vitro* and *in vivo*

**Figure 4:**
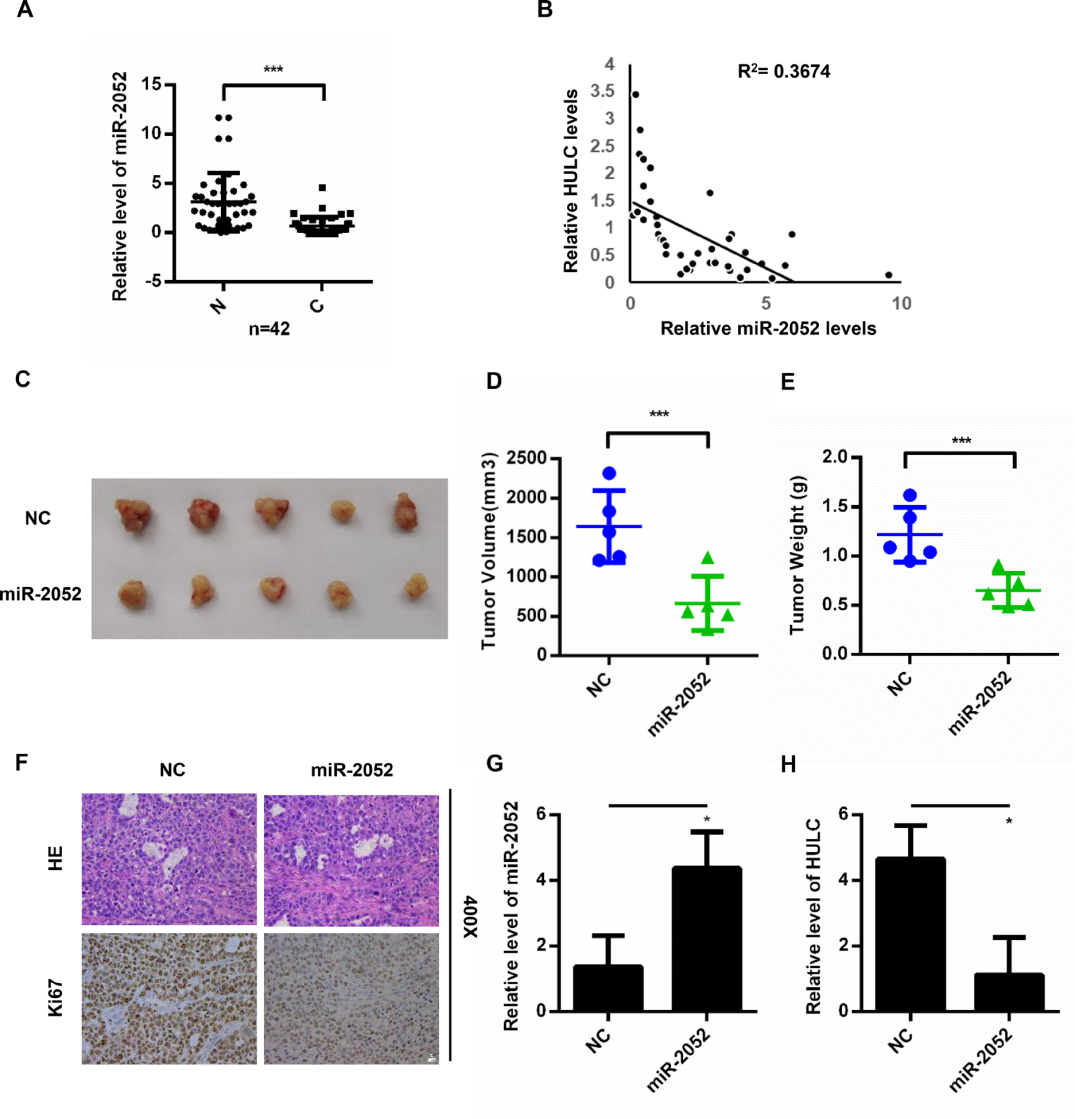
**miR-2052 inhibits the proliferation, migration, and invasion of HCC cells *in vitro* and *in vivo*.** (**A**) miR-2052 expression in HCC tissues analyzed by qPCR (n=42). (**B**) Correlation between HULC and miR-2052 expression in paired HCC tissues (n=42). (**C**–**E**) Tumor volume and weight measured in tissues from nude mice injected with miR-2052 stable HLF cells. (**F**) Ki67 levels measured by immunohistochemistry. (**G**, **H**) The expression of miR-2052 and HULC in xenograft tumors measured by qPCR. *P < 0.05, ***P < 0.001.

We next measured miR-2052 levels in 42 pairs of HCC and matched non-cancerous tissues. We found decreased miR-2052 levels in HCC tissues (Figure 4A), and miR-2052 expression correlated negatively with HULC expression (Figure 4B). Furthermore, results from CCK8 assays showed that miR-2052 inhibited the proliferation of HCC cells (Supplementary Figure 2A–2D). Additionally, trans-well assay showed that miR-2052 mimic reduced migration of HLF and 97H cells (Supplementary Figure 2E) while miR-2052 inhibitor increased it (Supplementary Figure 2F). Next, we used a xenograft tumor model to explore the role of miR-2052 *in vivo*. Compared with controls, the weights and volumes of tumors in the miR-2052 group were decreased (Figure 4C–4E). In addition, IHC showed that xenograft tumors expressing miR-2052 displayed lower Ki67 expression (Figure 4F). We then measured miR-2052 and HULC expressions in xenograft tumors by qPCR and found that the HULC expression was decreased while miR-2052 expression was increased (Figure 4G, 4H). These results demonstrated that miR-2052 inhibits the proliferation, migration, and invasion of HCC cells.

### MET is a direct target of miR-2052

**Figure 5:**
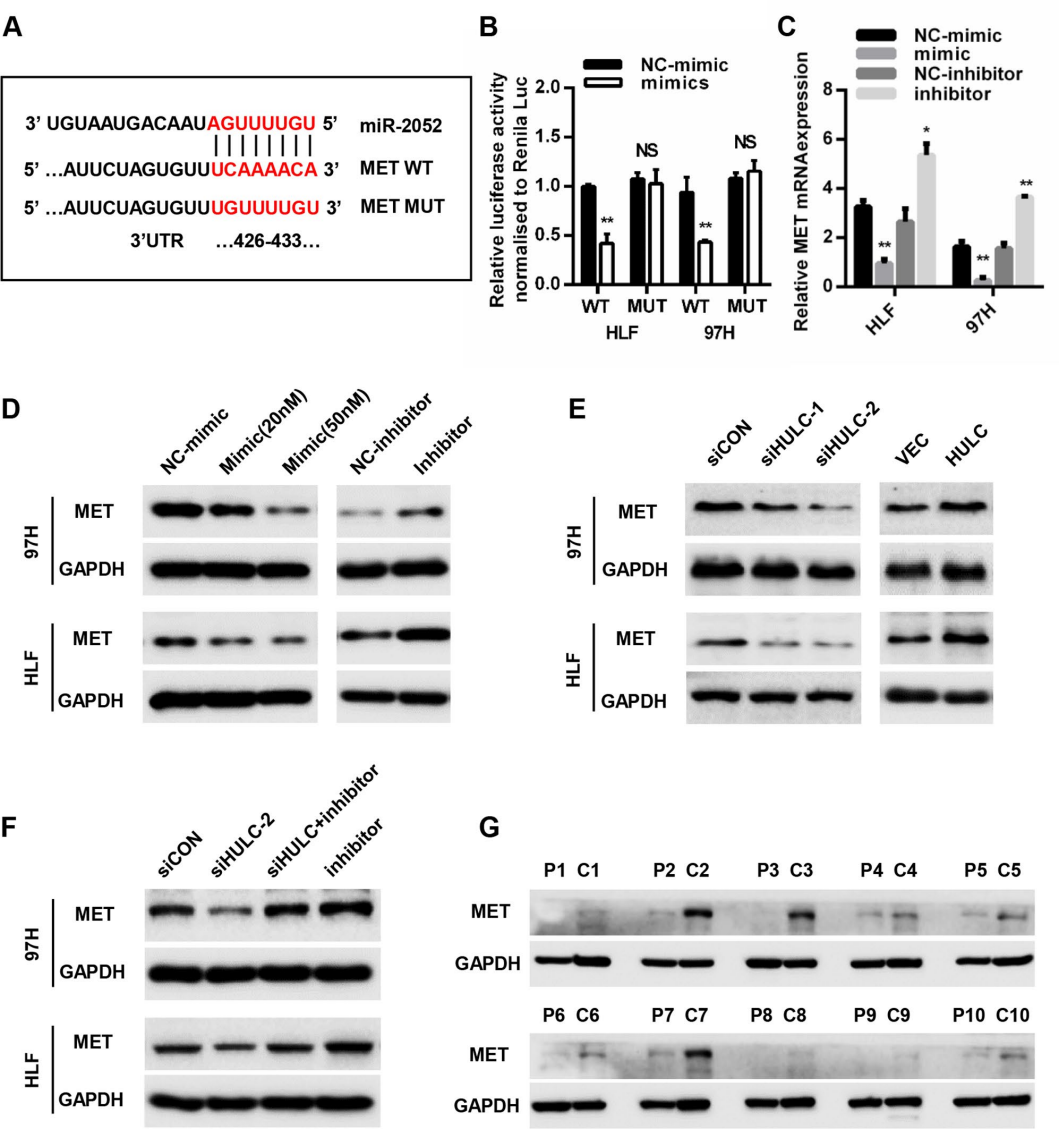
**MET is a direct target of miR-2052.** (**A**) Schematic view of miR-2052 putative binding site in the WT and MUT 3′ UTR of MET. (**B**) Luciferase activity assays in HCC cells transfected with WT and MUT 3′ UTR of MET luciferase reporter plasmids with miR-2052 mimics. (**C**, **D**) Relative mRNA and protein levels of MET in HLF and 97H cells after miR-2052 mimics and inhibitors transfection. (**E**) MET protein levels in HLF and 97H cells after HULC knockdown or overexpression. (**F**) MET protein levels in HCC cells after HULC knockdown with or without miR-2052 inhibition. (**G**) MET protein levels in HCC tissues (n=42). *P < 0.05, **P < 0.01.

To determine the target genes of miR-2052, we searched four online bioinformatics tools (MicroT, miRDB, miRWalk, and TargetScan) and jointly predicted that four genes may be biological targets of miR-2052 (Supplementary Figure 3A). We tested the regulation of these genes by miR-2052 through qPCR, and chose MET in the end as the focus of subsequent experiments (Supplementary Figure 3B–3C). To further clarify the relationship between miR-2052 and MET, we identified the potential binding sites between miR- 2052 and MET (Figure 5A). Luciferase reporter assay results showed that miR-2052 mimic suppressed the luciferase activity of the MET wild type (WT) reporter, but not that of mutant (MUT) reporter in HLF and 97H cells. Moreover, qPCR and western blot (WB) showed that miR-2052 mimic suppressed, and miR-2052 inhibitor promoted, the expression of MET, respectively (Figure 5C, 5D). These results suggested that MET is a target of miR-2052. Consistently, we found that knockdown of HULC reduced, whereas overexpression of HULC increased MET expression (Figure 5E). Furthermore, inhibition of miR-2052 rescued HULC silencing-mediated downregulation of MET (Figure 5F), indicating that HULC promotes MET expression through sponging miR-2052 in HCC. In addition, WB of 42 pairs of HCC and control tissues revealed that MET was upregulated in HCC tissues (Figure 5G and Supplementary Figure 3D, 3E). Taken together, these results demonstrated that MET is a direct target of miR-2052 in HCC cells.

### HULC promotes HCC progression through the miR-2052/MET axis *in vitro*

**Figure 6:**
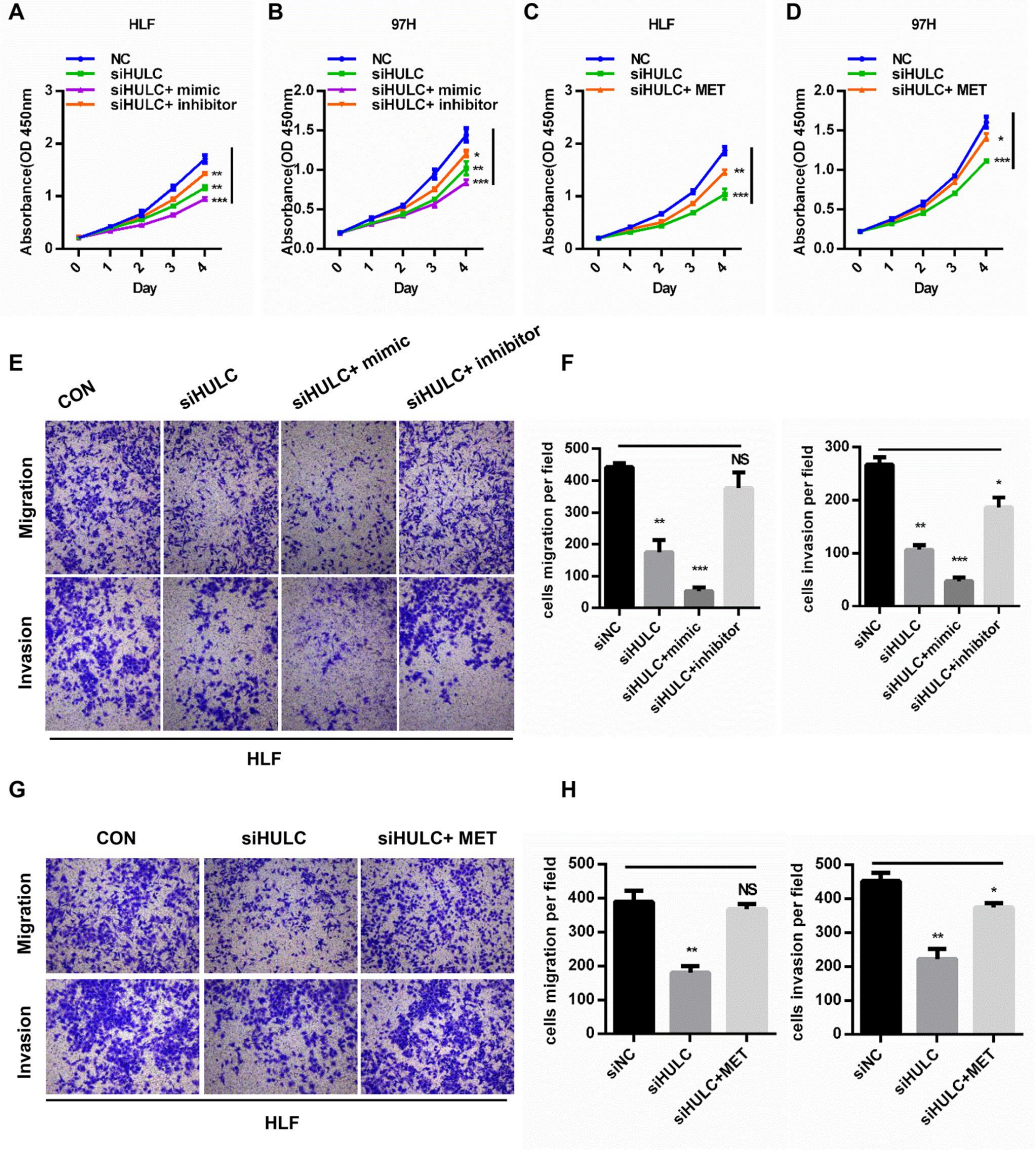
**HULC promotes HCC progression through the miR-2052/MET axis *in vitro*.** (**A**–**D**) CCK8 assays for HLF and 97H cells cotransfected with siHULC and miR-2052 mimic or inhibitor or MET. (**E**–**H**) Transwell assays for HLF and 97H cells cotransfected with siHULC and miR-2052 mimic or inhibitor or MET. *P < 0.05, **P < 0.01, ***P < 0.001.

To further test whether HULC promoted HCC progression by targeting the miR-2052/MET axis, we cotransfected miR-2052 mimic or inhibitor together with HULC silencing in HCC cells. CCK8 assays demonstrated that HULC knockdown inhibited the proliferation of HCC cells while restoration of miR-2052 mimic accentuated and restoration of miR-2052 inhibitor abrogated the suppressive effects of HULC silencing (Figure 6A, 6B). Consistently, restoration of MET abrogated the suppressive effects of HULC silencing (Figure 6C, 6D). Transwell migration and invasion assays yielded similar results (Figure 6E–6H). Further analysis showed MET protein expression correlated positively with HULC expression in HCC tissues (Supplementary Figure 3F). Taken together, these results demonstrated that HULC promotes HCC progression through the miR-2052/MET axis.

### HULC promotes HCC growth through miR-2052/MET axis *in vivo*

**Figure 7:**
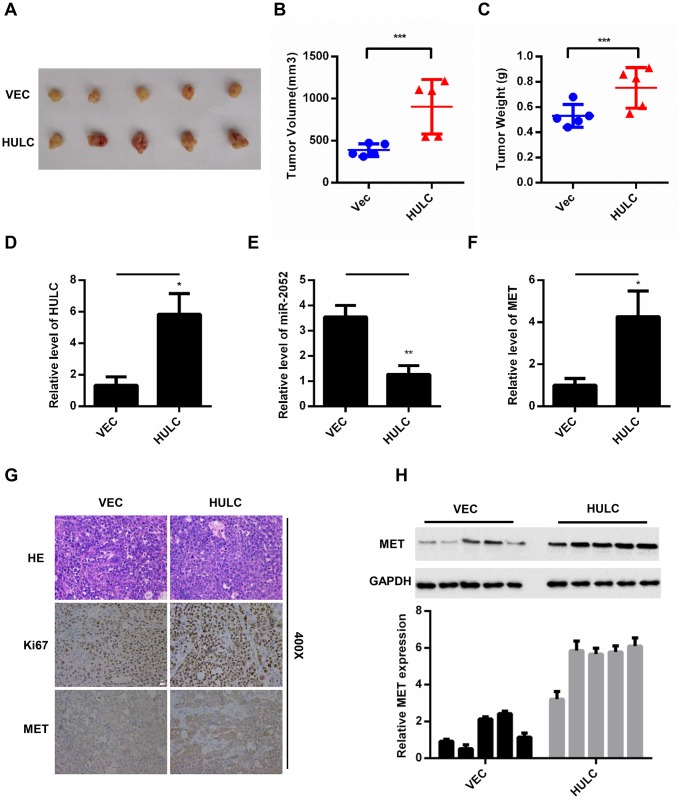
**HULC promotes HCC growth through the miR-2052/MET axis *in vivo*.** (**A**–**C**) Volume and weight measurements for tumors from nude mice injected with HLF cells stably overexpressing HULC. (**D**–**F**) Expression of HULC, miR-2052, and MET in tumor xenografts measured by qPCR. (**G**) Ki67 and MET levels measured by immunohistochemistry. (**H**) MET levels measured by western blot and gray level analysis. *P < 0.05, **P < 0.01, ***P < 0.001.

To further analyze the effect of HULC on HCC growth *in vivo*, we exploited the xenograft mouse model. HLF cells overexpressing HULC have a remarkably increased tumor volume and weight (Figure 7A–7C). In addition, HULC, miR-2052, and MET levels were measured by qPCR in xenograft tumors. The results showed that HULC and MET expression was increased, whereas miR-2052 expression was decreased (Figure 7D–7F). Then, IHC results showed that xenograft tumors overexpressing HULC displayed high Ki67 and MET expression (Figure 7G). Moreover, we also measured MET expression in xenograft tumors by WB and the results showed that MET was increased in cells overexpressing HULC (Figure 7H). Taken together, these results indicated that HULC promotes HCC growth through the miR-2052/MET axis *in vivo*.

## DISCUSSION

It is known that HULC is an oncogenic noncoding RNA. In the current study, we investigated the mechanisms by which HULC contributes to HCC development. Here, we found that HULC was overexpressed in HCC tissues, and increased HULC levels correlated positively with poor prognosis of HCC patients. Through HULC interference and overexpression experiments, we demonstrated that HULC plays a promoter role in the development of HCC both *in vitro* and *in vivo*. Mechanistically, high HULC levels upregulated MET expression through miR-2052 ceRNA mechanism (Supplementary Figure 4A). Thus, our study highlights the importance of the miR-2052/MET axis to the oncogenic function of HULC in HCC.

HULC was the first identified lncRNA that was specifically overexpressed in liver cancer [19]. The Matouka group demonstrated that HULC is upregulated in cell lines producing HBV [21]. Du et al. showed that upregulation of HULC by HBx promotes the proliferation of hepatoma cells by downregulating the tumor suppressor gene p18 [17]. Wang and colleagues further identified a regulatory loop between HULC and CREB, in which HULC may act as an endogenous “sponge”, which downregulates miR-372 to reduce the translational repression of its target gene, PRKACB, which in turn induces phosphorylation of CREB [15]. Previous studies have shown that HULC silencing inhibits the growth of gastric cancer cells and weakens their drug resistance [22]. HULC is known to promote multiple types of cancer [8, 23–25] and to be overexpressed in HCC [26], with such overexpression being an independent unfavorable factor for overall survival and disease-free survival time in HCC patients [27]. HULC was able to elevate HBx, which coactivated STAT3 to stimulate the miR-539 promoter in HBV-related HCC [28]. Recently, HULC can be used as a potential cancer biomarker for the diagnosis of HCC [29–31]. In addition, Zhao et al*.* found that circulation of HULC was increased in plasma samples of HBV-related cirrhosis patients [32]. In agreement with these previous studies, our results also revealed that HULC overexpression contributed to the development of HCC.

The role of miR-2052 in HCC cells has not been reported. Our results demonstrated that the expression of miR-2052 was lower in HCC tissue than that in adjacent normal controls, and that miR-2052 inhibited the proliferation and metastasis of liver cancer cells, suggesting that miR-2052 may be a tumor suppressor in HCC. We also performed bioinformatics analyses to find potential targets for miR-2052, from which MET emerged as a likely candidate. We then demonstrated that miR-2052 directly targets MET and found that HULC promoted MET expression by inhibiting miR-2052. In addition, MET was also upregulated in HCC tissues, suggesting that the HULC/miR-2052/MET axis may be involved in the development of HCC. MET is a pivotal tyrosine kinase that promotes tumorigenesis and tumor metastasis. Indeed, previous studies have reported that MET promotes proliferation, motility, drug resistance, and the epithelial–mesenchymal transition of cancer cells [33]. Ponzo MG et al. reported that MET contributed to endothelial cell growth, invasion, and motility in breast cancer [34]. MET is overexpressed in osteosarcoma tissues and facilitates osteosarcoma cell proliferation, migration, and invasion [35]. In one recent study, NEAT1 suppressed miR-335 expression and facilitated tumor growth and invasion via disinhibition of MET [36]. These results are largely in line with our observations here on HCC models. Our data also showed that MET was upregulated in HCC tissues and that MET partially reverses the knockdown effect of HULC, suggesting that the inhibitory effect of HULC knockdown on HCC cells is dependent on the inhibition of MET expression.

In conclusion, our study found that HULC promotes the progression of liver cancer via the HULC/miR-2052/MET axis, providing a potential diagnostic and therapeutic target for HCC.

## MATERIALS AND METHODS

### Patients and tissue specimens

A total of 42 pairs of randomly selected snap-frozen samples from consecutive patients who received hepatectomy for HCC at the Hepatic Surgery Center, Tongji Hospital of Huazhong University of Science and Technology (HUST) (Wuhan, China) from January 2012 to December 2014 constituted the training cohort. The inclusion criteria (diagnosis status) of all patients was confirmed by two independent histopathologists; The surgical indication was an HCC patient with liver tumor of enough residual liver volume, and lack of distant metastasis, decompensated cirrhosis, and organic dysfunction. All research on human materials was approved by the Ethic Committee of Tongji Hospital, Huazhong University of Science and Technology and the study was conducted according to the Declaration of Helsinki. Written informed consent was obtained from each patient.

### Cell lines and cell culture

HCC cell lines 97H and LM3 were purchased from the Liver Cancer Institute of Fudan University. The 7702, HepG2, Huh7, Hep3B and ALEX were purchased from China Center for Type Culture Collection (CCTCC, Wuhan, China). HLF and SK-hep1 cells were deposited in the Hepatic Surgery Center, Tongji Hospital. These cell lines were cultured in Dulbecco’s modified Eagle’s medium (Invitrogen) supplemented with 10% fetal bovine serum (Gibco, Grand Island, NY) and incubated in 5% CO_2_ at 37 °C.

### Plasmid construction, cell transfection and RNA immunoprecipitation

The full length of the HULC gene was synthesized by Tsingke Biological Technology (Beijing, China), and sub-cloned into the BamHI/EcoRI sites of pCDNA3.1 vector to generate the pCDNA3.1-HULC, and the empty pCDNA3.1 was used as control. HULC was also sub-cloned into pcDNA3.1 vector with 6×MS2 stem-loop. The MS2 coding sequence was sub-cloned into pcDNA3.1-GST vector to get the pEGST-MS2 construct. All small RNA molecules were ordered from RiboBio (Guangzhou, China), including miR-2052 mimics (UGUUUUGAUAACAGUAAUGU), mimics negative controls (mimics-NC) (UUUGUAC UACACAAAAGUACUG), miR-2052 inhibitor (miRNA inhibitors are chemically modified antisense oligonucleotides with increased stability and activity, and are a complement to corresponding mature miRNA sequences, used in miRNA loss-of-function studies), inhibitor negative controls (inhibitor-NC), and small interfering RNAs (siRNAs) against HULC. Lentivirus particles containing HULC and miR-2052 were ordered from GeneChem (Shanghai, China). HLF and 97H cell transfection was carried out using Lipofectamine® 2000 (Invitrogen) following the manufacturer’s instructions. After culturing for 48 h, the cells were harvested, and transfection efficiency was tested by qPCR. HLF and 97H cells were cotransfected with pcDNA3.1-MS2-HULC and pEGST-MS2. After 48 h, cells were used to perform RNA immunoprecipitation (RIP) experiments using GST or IgG antibody (Sigma) and the Imprint®RNA Immunoprecipitation (RIP) Kit (Sigma) according to the manufacturer’s instructions. The miRNA in the RIP material was detected by qPCR analysis.

### Cell proliferation assay

Before analysis of cell proliferation, HLF and 97H cells were seeded into 96-well plates at a concentration of 1000 cells/well. Then proliferation was determined using the Cell Counting Kit-8 (CCK-8, Dojindo, Tokyo, Japan) according to the manufacturer’s protocol. All experiments were performed three times and the average percentages of cells are shown.

### Transwell cell migration and invasion assays

Cell migration assays were performed using a 24-well Transwell plate (pore size, 8 μm; Corning, NY, USA), according to the manufacturer's protocol. For the Matrigel invasion assay, filters were precoated with 40 μl 1:4 mixture of Matrigel (BD Biosciences, NJ, USA) and DMEM for 4 h at room temperature. Briefly, for invasion and migration assays, culture medium containing 10% FBS was added to the lower chambers and aliquots of 5x104 cells in 100 μl serum-free medium were seeded into the upper chambers. After a 24 h incubation at 37 °C, non-migrated or non-invaded cells were removed by scraping the upper surface of the membranes with a cotton swab. Cells on the lower surface of the membranes were fixed with 4% paraformaldehyde at room temperature for 15 min and stained with 0.1% crystal violet at room temperature for 20 min. Cell numbers were counted under an optical microscope. Each experiment was repeated at least three times.

### qRT–PCR

TRIzol reagent (Invitrogen) was used to extract the total RNA from tissues and cells according to a modified version of the manufacturer’s protocol. The reverse transcription of lincRNA and mRNA was completed using a reverse-transcription system kit (Takara, Otsu, Japan) and assayed by qPCR using a standard SYBR Green PCR kit (Toyobo Life Science, Osaka, Japan) according to the manufacturers' protocols with GAPDH as the endogenous control to measure mRNA levels. Reverse transcription of miRNA was performed using an miRNA First-Strand cDNA Kit (TIANGEN, Beijing, China) and assayed by qPCR using an miRcute Plus miRNA qPCR Kit (TIANGEN, Beijing, China) with U6 as the endogenous control for miRNA expression. Relative quantification was performed using the comparative CT (2^-ΔΔCT^) method. Each assay was repeated three times, independently of each other.

### Luciferase assays

The entire 3’-untranslated region (UTR) of the MET and the full length of HULC gene were cloned into the psiCHECKTM-2-vector (Promega, Madison, WI, USA) at a site immediately downstream of the Renilla luciferase gene. The mutations in the 3′-UTR and HULC binding sites were generated with the Quick Change Site-Directed Mutagenesis kit (Stratagene, CA, USA). About 1 × 105 cells/well were seeded into 24-well plates for 24 h before transfection. Cells were cotransfected with 50 ng of the psiCHECKTM-2-vector and 50 nM of the miR-2052 or mimic-NC using Lipofectamine 2000 (Invitrogen). Cell lysates were prepared using Passive Lysis Buffer (Promega) 48 h after transfection, and luciferase activity was measured using the Dual-Luciferase Reporter Assay (Promega). Experiments were repeated three times.

### Western blot

Cells were lysed with RIPA buffer containing a protease inhibitor cocktail (Roche) on ice for 30 min. Cell lysates were quantified using a BCA Protein Assay Kit (Promega) and equal amounts (20 μg/lane) of protein were analyzed by 10% SDS-PAGE and transfer to polyvinylidene fluoride membranes (Roche). The membranes were blocked with 5% non-fat milk at 37 °C for 1 h and were incubated with primary antibodies at 4 °C overnight. Subsequently, the membranes were incubated with HRP-conjugated goat anti-rabbit or goat anti-mouse immunoglobulin G secondary antibodies (Jackson ImmunoResearch Laboratories) for at 37 °C for 1 h. Finally, the enhanced chemiluminescence detection system (Bio-Rad Laboratories) was used for visualization. Image Lab™ 4.0 software (Bio-Rad Laboratories) was used to semi-quantify blots. MET antibody was purchased from CST (#8198; Cell Signaling Technology, USA).

### Immunohistochemistry

Tumor xenografts were fixed in 4% neutral formalin at room temperature for 24 h, embedded in paraffin and cut into 4 μm sections. The sections were then deparaffinized in xylene, rehydrated in a graded alcohol series and treated with boiling 0.01 mol/l citrate buffer for 15 min for antigen retrieval. Endogenous peroxidase activity was blocked with hydrogen peroxide (0.3%) at room temperature for 15 min, and the sections were incubated with 5% bovine serum albumin (R&D Systems, Minneapolis, MN, USA) at 37 °C for 45 min to reduce non-specific binding. Immunostaining with MET antibody (#8198; Cell Signaling Technology) or Ki-67 (ab15580; Abcam, Cambridge, UK) were carried out at 4 °C for 16 h, followed by incubation with a horseradish peroxidase (HRP) -conjugated secondary antibody from the Envision kit (Dako; Agilent Technologies, Inc., Santa Clara, CA, USA) for 45 min at room temperature. Antibody binding was detected by DAB (Dako; Agilent Technologies, Inc.), according to manufacturer's protocol, at room temperature for 1 min and the reaction was terminated by immersion of tissue sections in distilled water once brown staining appeared. Tissue sections were counterstained with 1% hematoxylin at room temperature for 3 min and dehydrated in a graded series of ethanol. Images of representative fields were obtained from Nikon Digital ECLIPSE C1 microscope (Nikon Corporation).

### Xenograft mouse model

A total of 20 male BALB/c nude mice (5-week old) were divided into four groups (NC, miR-2052 and VEC, HULC). Then, 2 x 10^6^ logarithmically growing HLF cells stably expressing NC, miR-2052, VEC, and HULC were subcutaneously injected into nude mice. After 5 weeks, the nude mice were killed, and the tumor tissues were stripped and weighed. Tumor volume was calculated using the formula: V (mm^3^) = 0.5 x L (mm) x W2 (mm^2^). Total RNA and protein were extracted from the tissues, and the expression of HULC, miR-2052, and MET was measured by qPCR or WB.

### Statistical analysis

All statistical analyses were performed using SPSS 21.0 statistical software. Data were compared using Student’s two-tailed test. Data are represented as the mean ±SEM. Analysis of variance (ANOVA) was performed to determine statistically significant differences. A value of P < 0.05 was considered statistically significant, *, P< 0.05; **, P< 0.01; ***, P< 0.001.

## Supplementary Material

Supplementary Figures
