# Supplementary material for: Long noncoding RNA HULC promotes hepatocellular carcinoma progression
Source: Aging (Albany NY). 2019 Oct 23;11(20):9111–27. doi: 10.18632/aging.102378 (PMC6834430; doi:10.18632/aging.102378)
Supplement: Supplementary Figures [file aging-11-102378-s001.pdf]

SUPPLEMENTARY FIGURES

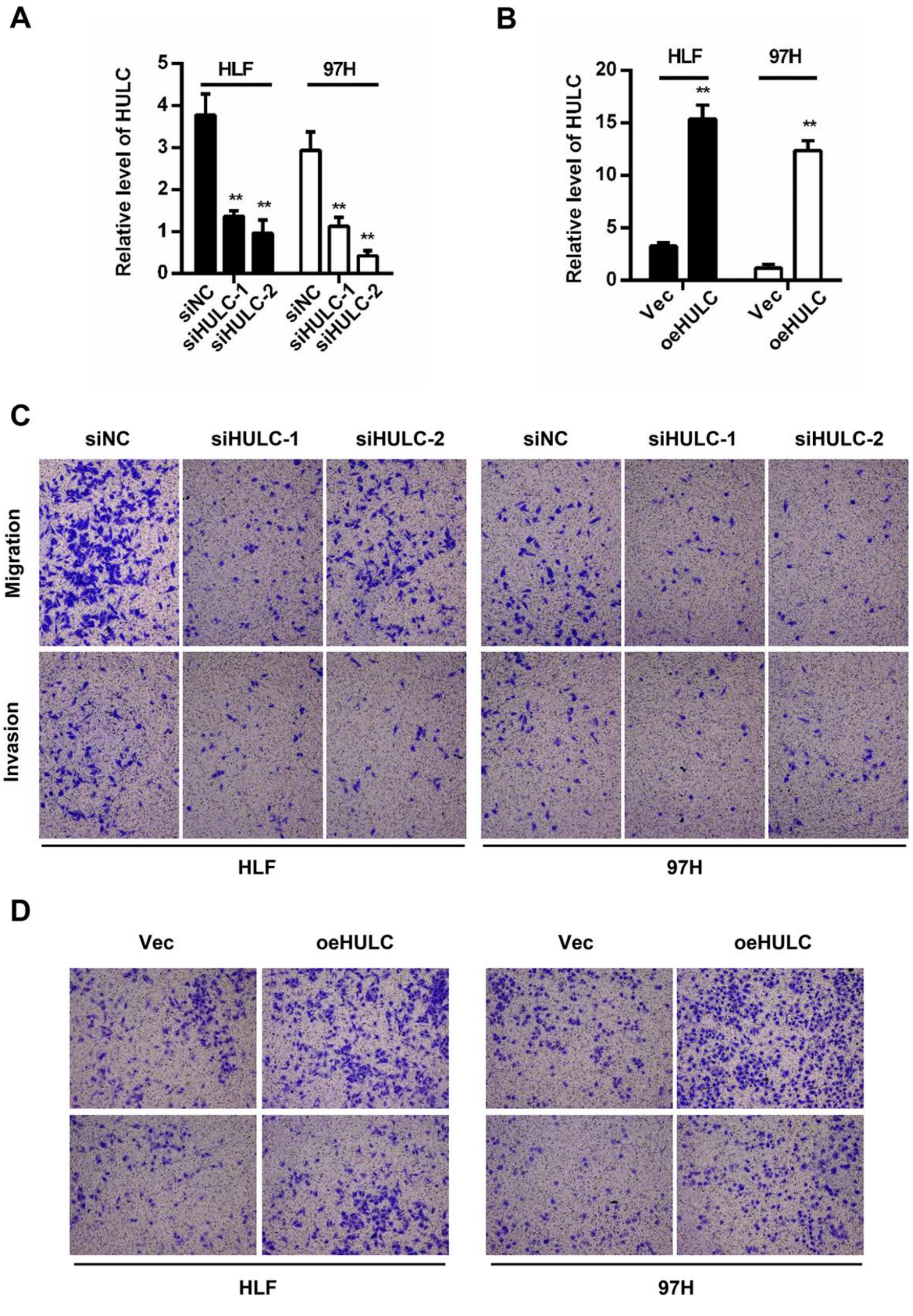

Supplementary Figure 1. HULC promotes HCC cells migration, and invasion. Quantitative PCR showing that HULC levels were (**A**) downregulated after siHULC transfection and (**B**) upregulated after pcDNA-HULC transfection. (**C**, **D**) Representative images of transwell assay. **P < 0.01.

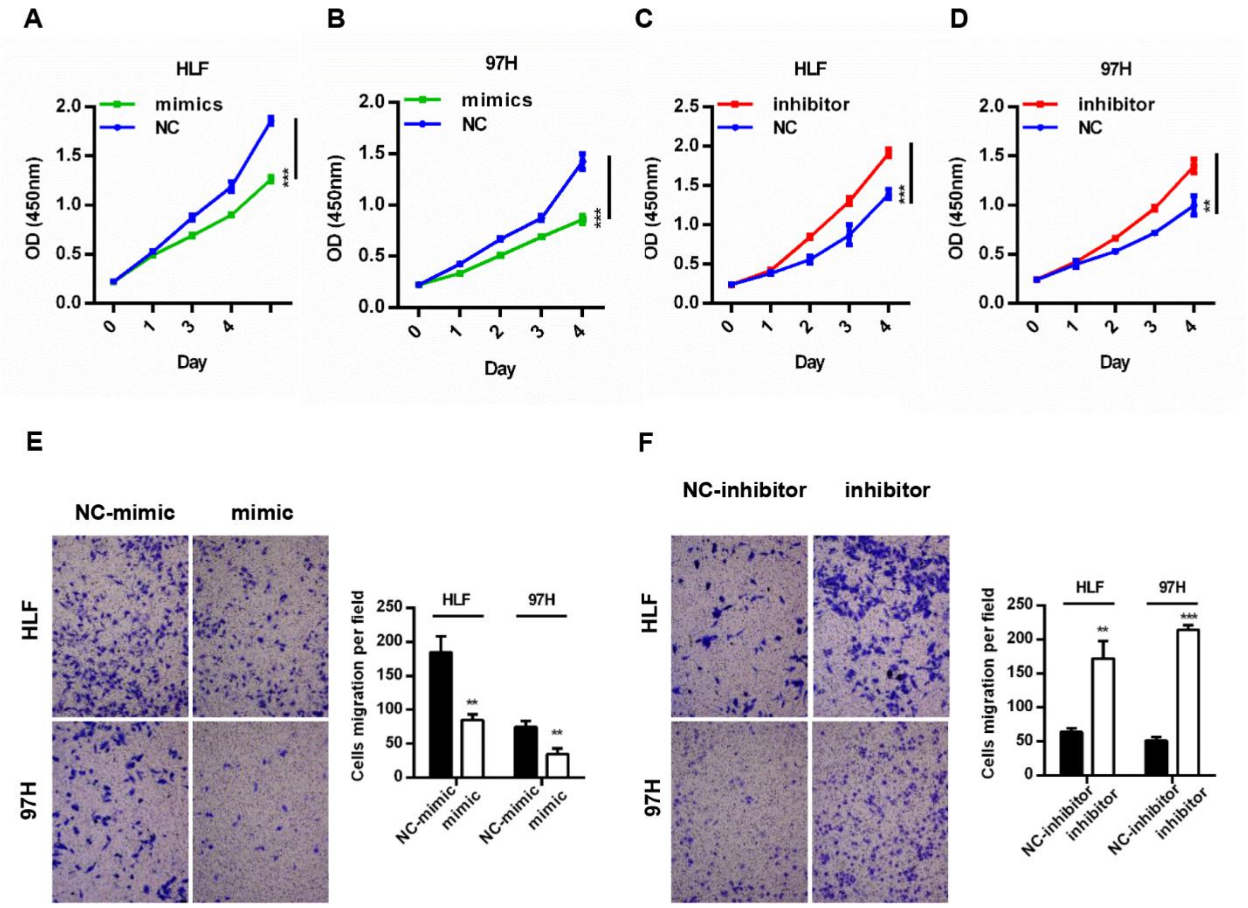

Supplementary Figure 2. miR-2052 inhibits HCC cells proliferation and migration. (**A**–**D**) Viability of HCC cells transfected with miR-2052 mimic or inhibitor measured by CCK8 assays. (**E**–**F**) Transwell assay showing that miR-2052 suppressed HCC cell migration. **P < 0.01, ***P < 0.001.

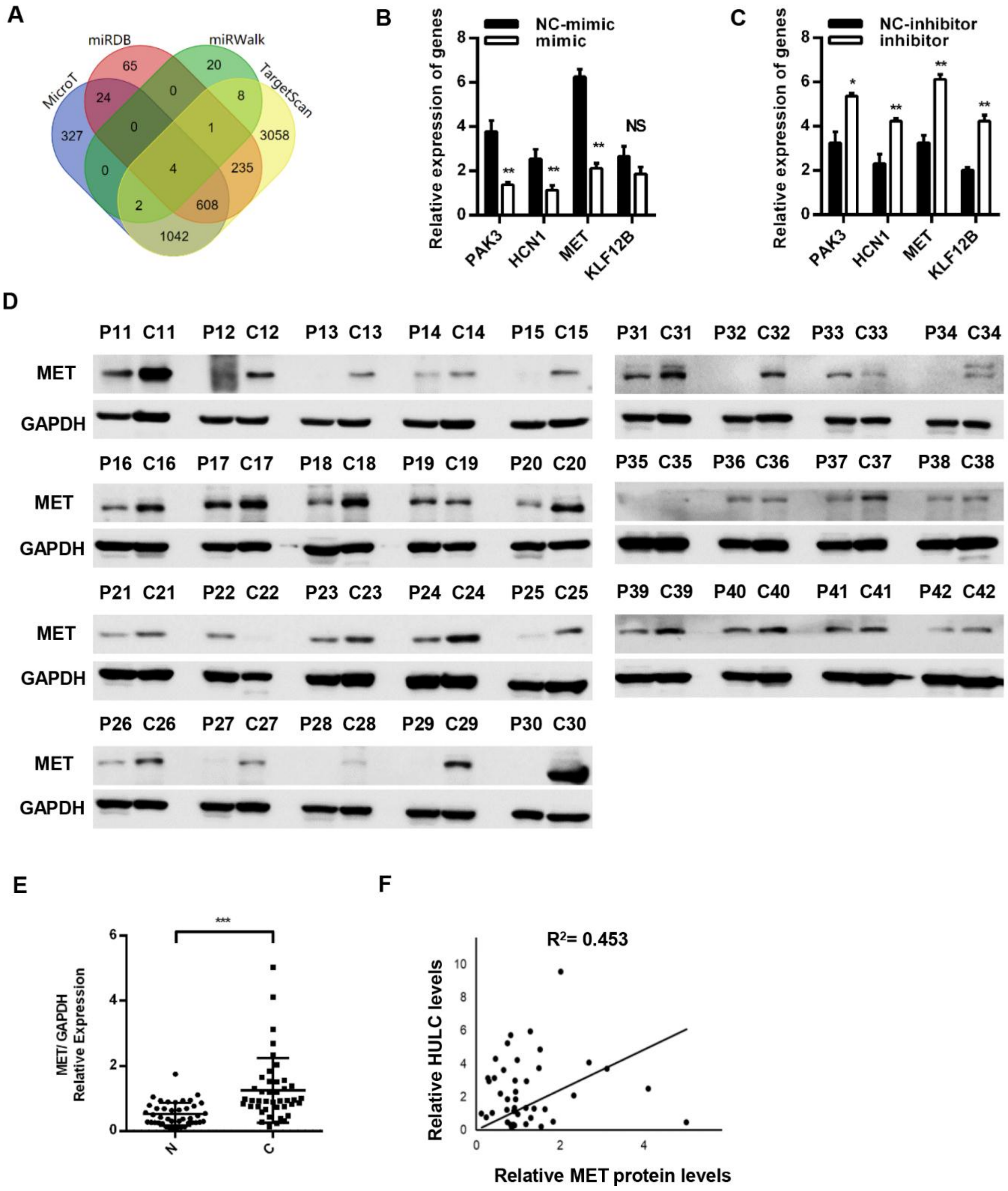

Supplementary Figure 3. MET is a direct target of miR-2052. (**A**) Venn diagrams showing the number of potential mRNAs targeted by miR-2052. (**B**, **C**) qPCR analysis of the expression of four candidate mRNAs when treated with miR-2052 mimic or inhibitor. (**D**) MET protein levels in HCC tissues, and (**E**) statistical analysis of relative MET levels in HCC tissues compared to normal tissue controls (n=42). (**F**) Correlation between HULC and MET expression in paired HCC tissues (n=42). *$P < 0.05$, **$P < 0.01$, ***$P < 0.001$.

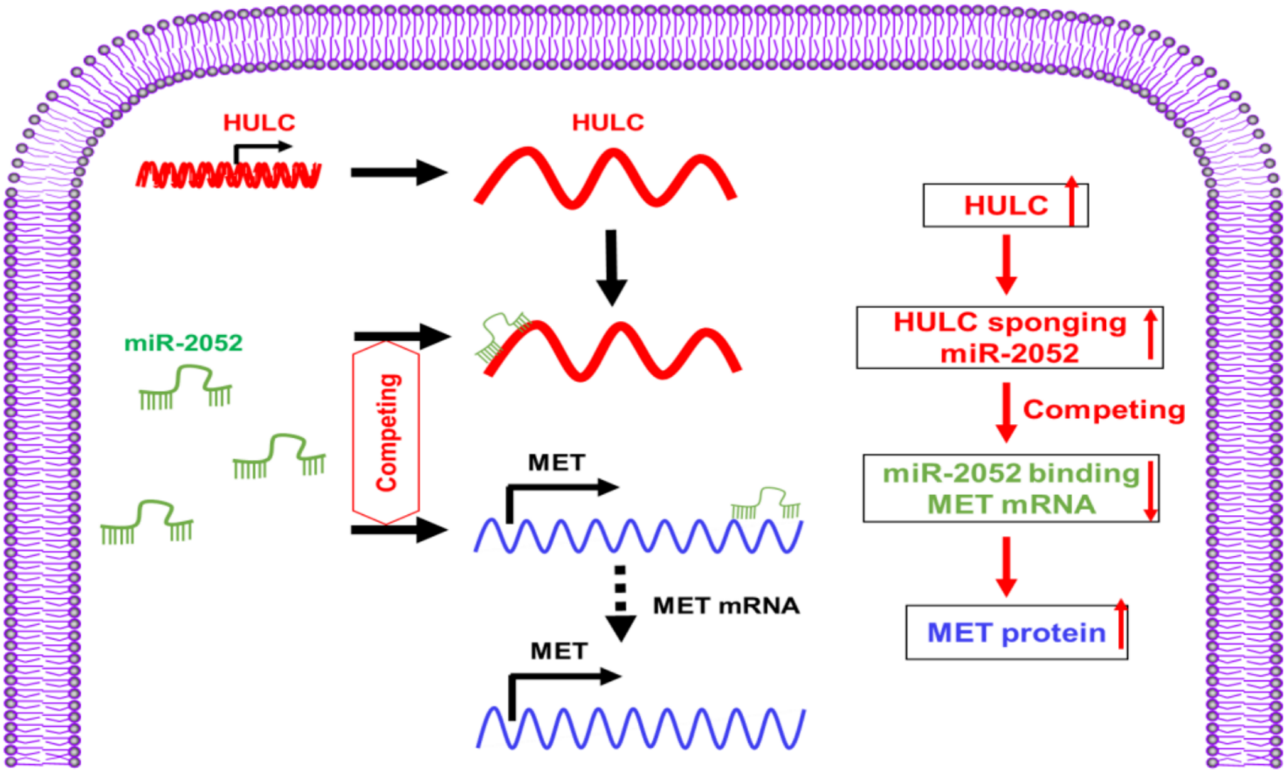

Supplementary Figure 4. A schematic figure of the HULC/miR-2052/MET axis. (**A**) A schematic figure demonstrates the regulatory mechanism of the HULC/miR-2052/MET axis.
